# Title Corrections: Erratum

**DOI:** 10.1097/MD.0000000000024964

**Published:** 2021-02-26

In recent issues, *Medicine* has published two^[1,2]^ articles with protocol mistakenly added to the title. The titles have been updated to remove this.
